# Novel Antithrombotic Agents in Ischemic Cardiovascular Disease: Progress in the Search for the Optimal Treatment

**DOI:** 10.3390/jcdd9110397

**Published:** 2022-11-16

**Authors:** Ignacio Barriuso, Fernando Worner, Gemma Vilahur

**Affiliations:** 1 Hospital Universitario Arnau de Vilanova, Institut de Recerca Biomèdica de Lleida, 25198 Lleida, Spain; 2 Institut de Recerca, Hospital Santa Creu i Sant Pau, IIB Sant Pau, 08025 Barcelona, Spain; 3 Department of Medicine, Autonomous University of Barcelona, 08193 Barcelona, Spain; 4 Centro de Investigaciones Biomédicas En Red de enfermedades CardioVasculares (CiberCV), 28029 Madrid, Spain

**Keywords:** cardiovascular diseases, novel antithrombotic agents, antiplatelet drugs, anticoagulants, hemostasis

## Abstract

Ischemic cardiovascular diseases have a high incidence and high mortality worldwide. Therapeutic advances in the last decades have reduced cardiovascular mortality, with antithrombotic therapy being the cornerstone of medical treatment. Yet, currently used antithrombotic agents carry an inherent risk of bleeding associated with adverse cardiovascular outcomes and mortality. Advances in understanding the pathophysiology of thrombus formation have led to the discovery of new targets and the development of new anticoagulants and antiplatelet agents aimed at preventing thrombus stabilization and growth while preserving hemostasis. In the following review, we will comment on the key limitation of the currently used antithrombotic regimes in ischemic heart disease and ischemic stroke and provide an in-depth and state-of-the-art overview of the emerging anticoagulant and antiplatelet agents in the pipeline with the potential to improve clinical outcomes.

## 1. Introduction

Cardiovascular diseases (CVDs) remain the leading cause of death worldwide. In 2016, 17.9 million people died from all causes of CVDs [1,2]. There were approximately 8.9 million deaths due to ischemic heart disease (IHD) worldwide, remaining the first cause of death; a less prevalent disease was ischemic stroke, with an incidence of 7.6 million globally [3,4,5].

**Figure 1:**
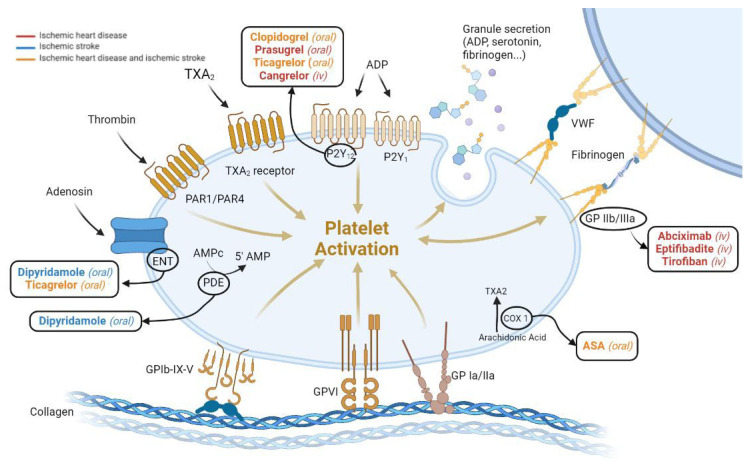
Antiplatelet drugs currently used to treat ischemic heart disease and ischemic stroke. ENT: equilibrative nucleoside transporter; ASA: acetylsalicylic acid; TXA2: thromboxane A2; VWF: Von Willebrand Factor; PDR: phosphodiesterase; COX: cyclooxygenase. Figure created with BioRender.com.

**Figure 2:**
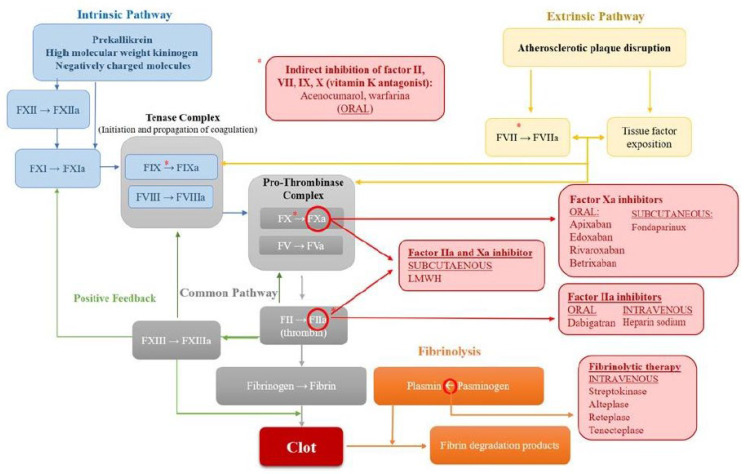
Diagram of the coagulation and fibrinolytic pathways and the different anticoagulants and fibrinolytic agents used in the clinical setting. LMWH: low molecular weight heparin. * Indirect inhibition of factors II, VII, IX and X.

Therapeutic advances in the last decades have reduced CVD mortality, with antithrombotic therapy being the cornerstone of medical treatment. Several antithrombotic drugs are currently used to either block platelet activation (Figure 1), prevent the activation of the coagulation cascade, or induce fibrinolysis once the clot is formed (Figure 2) [6,7,8,9]. Yet, although these antithrombotic agents have robustly demonstrated their effectiveness in preventing atherothrombotic events, they also carry an inherent risk of bleeding. Bleeding is associated with adverse cardiovascular outcomes and mortality; hence, there is a need to discover new targets and develop novel antithrombotic strategies to effectively inhibit thrombosis while preserving hemostasis.

In the following review, we will comment on the key limitations of the currently used antithrombotic regimes in ischemic heart disease and ischemic stroke and provide an in-depth and state-of-the-art overview of the emerging anticoagulant and antiplatelet agents in the pipeline with the potential to improve clinical outcomes.

## 2. The Coagulation Cascade: Targeting the Intrinsic Coagulation Pathway

Anticoagulants are the treatment of choice to prevent cardioembolic stroke in patients with atrial fibrillation [10,11]. During the last decade, the development of direct oral anticoagulants (DOACs; Figure 2) has brought many advantages as compared to vitamin K antagonists, including a predictable pharmacokinetic profile, rapid onset and offset of action, and fixed dosing with no need for laboratory monitoring or dietary discretion [12]. Conversely, different reversal agents have also been developed to block the effect of anticoagulants in case of need (Table 1).

Yet, important challenges still need to be addressed. As such, bleeding remains the most reported side effect of DOACs, and in certain sub-groups of patients, including patients with mechanical heart valves or triple-positive antiphospholipid disease syndrome, DOACs seem to be less effective than vitamin K antagonists and are not recommended [20,21,22].

Anticoagulants are also implemented in ischemic heart disease since patients who suffer an acute coronary event present an excess of thrombin generation that persists beyond the acute presentation [23]. So far, several trials have demonstrated the ability of anticoagulants to protect against cardiovascular events. As such, the addition of warfarin [24], rivaroxaban [25,26], or ximelagatran [27] to a standard antiplatelet regime has shown to significantly reduce ischemic events, though at the expense of increased bleeding risk. Ximelagatran was, however, withdrawn from the market due to hepatotoxicity and the only anticoagulant recommended by the guidelines for long-term secondary prevention is rivaroxaban, which may be administered at low doses on top of aspirin at 1-year post-MI [25].

Altogether, these trials have evidenced the need to discover new targets that effectively block thrombin generation without displaying hemorrhagic side effects. In recent years, special attention has focused on the main components of the intrinsic coagulation pathway, particularly factor (F)XII, FXI, and FIX [28].

### 2.1. Targeting Factor XII

FXII has been associated with thrombosis, hereditary angioedema, and (neuro) inflammation. On the other hand, FXII deficiency (i.e., Hageman factor deficiency) is a rare genetic blood disorder that is entirely asymptomatic, showing prolonged active partial thromboplastin times (aPTT) as the only alteration on coagulation tests [29]. FXII circulates in plasma in a zymogen form, and its activation is brought about by the interaction with negatively charged molecules that induce a conformational change in zymogen FXII leading to activated protease FXIIa followed by activation of the enzyme precursors FXI and FIX (Figure 2) [30]. Hence, FXII inhibitors are expected to be particularly efficient in patients whose blood is exposed to non-physiological surfaces of medical devices such as vascular catheters, hemodialysis circuit tubes and membranes, and mechanical valves or stents, that expose negative charge molecules [31]. Alternatively, contact system proteins FXII, high-molecular-weight kininogen (HK), and plasma kallikrein (PK) may assemble on cell surface proteoglycans of various cardiovascular cells. Contact with surface-exposed moieties and plasma-borne soluble contact activators induces FXII activation, which initiates the intrinsic coagulation pathway and activates PK leading to the release of the proinflammatory mediator bradykinin (BK) by PK-mediated cleavage of HK. FXII inhibitors are also being evaluated as a potential treatment for hereditary angioedema, a BK-mediated life-threatening inherited swelling disorder where Serpin C1 esterase inhibitor (a major plasma inhibitor of FXII and and PK) is dysfunctional or deficient [16].

Multiple prototypes have been discovered within the last years, including monoclonal antibodies, small interfering RNAs, antisense oligonucleotides, and serine protease inhibitors which are currently being tested at a preclinical level (details are provided in Table 2) [32]. However, each one of these strategies confers different pharmacological properties, which may limit their indications. Antibodies and approaches to silence gene expression require parenteral delivery by subcutaneous or intravenous injection, whereas small molecules can be delivered orally or parenterally (Table 2). On the other hand, small interfering RNAs and antisense oligonucleotides have a slow onset of action requiring about four weeks to achieve therapeutic levels. Although they are not optimal for use in acute settings, their effect extends over time which may enable once-monthly administration. On the other hand, however, they may also require the development of antidotes. In contrast, antibodies and serine protease inhibitors have a rapid onset of action and an expected half-live of <24 h, thereby limiting the need to develop reversal strategies [33].

Garadacimab, a monoclonal antibody, has been the sole FXIIa inhibitor to reach phase III clinical trials (NCT04656418) in patients with hereditary angioedema, showing promising preliminary data after a 6-month follow-up. Another phase III trial (NCT04739059) is ongoing to evaluate its benefits in a longer term (32 months). Based on its proven safety profile and the outcome of both trials, garadacimab may be considered a promising strategy for other indications, including CVDs.

### 2.2. Targeting Factor XI

Factor XI congenital deficiency has been proven to protect against arterial and venous thrombosis reducing the incidence of deep-vein thrombosis, ischemic stroke, myocardial infarction, and vascular graft occlusion [48,49,50,51]. Most importantly, FXI-deficient patients do not generally exhibit spontaneous bleeding, and the bleeding associated with injury or surgery tends to be mild [52,53]. These observations have supported the development of multiple FXI inhibitors, most of which have reached Phase II testing. Table 3 details the studies conducted so far as per FXI inhibitors.

### 2.3. Targeting Factor IX

Factor IX is another potential drug target currently under intensive research because of its efficacy and safety profile [72]. Factor IX is activated by both the intrinsic and extrinsic pathways (Figure 2). In the intrinsic pathway, FXIa induces FIX activation, whereas, in the extrinsic coagulation pathway, FIX is activated by the tissue factor (TF)–VIIa complex. FIXa forms a complex with FVIIIa that binds to platelets serving as a very potent activator of FX [73].

As for FXIIa and FXIa, multiple FIX inhibitors have been developed, most of them in the preclinical development phase, and only a few have reached clinical trials (Table 4) [74]. One that raised great interest is pegnivacogin, a RNA-aptamer based FIXa inhibitor featuring a reversal agent, anivamersen [73]. However, both phase II trials where pegnivacogin has been tested have not resulted in the expected positive outcome. The RADAR trial in NSTEMI patients undergoing cardiac catheterization [75] did not show differences between pegnivacogin and heparin, and the REGULATE-PCI trial performed in patients undergoing PCI [76] had to be prematurely terminated due to the presence of severe allergic reactions.

## 3. Targeting the Platelet: What Is in the Pipeline for Novel Antiplatelet Agents?

Antiplatelet agents are used in treating both ischemic heart disease and ischemic stroke (Figure 1). The currently available antiplatelet agents either: (1) target intraplatelet enzymes (COX-1 inhibition by ASA and PDE inhibition by dipyridamole and cilostazol), preventing the formation of thromboxane A2 (TXA_2_) or the degradation of AMPc, respectively; or (2) block platelet membrane receptors (P2Y_12_ receptor antagonists, GPIIb/IIIa inhibitors, and PAR antagonist) preventing their downstream signaling activation (Figure 1) [80].

In the setting of ischemic heart disease, antiplatelet agents are used both in acute and chronic coronary syndromes and after stent implantation to prevent stent-related thrombosis [81,82,83,84]. A double antiplatelet regime with a combination of ASA and a P2Y_12_ inhibitor is recommended during the first year after an acute myocardial event [85,86,87]. Among the P2Y_12_ inhibitors, clopidogrel, a second-generation thienopyridine, is a pro-drug that requires a two-enzyme-mediated transformation to become active and irreversibly block the P2Y_12_ platelet ADP receptors. Yet, clopidogrel exhibits high individual variability because of differences in the activity of cytochrome P450 2C19. Prasugrel, a third-generation thienopyridine, is also a pro-drug but requires fewer hepatic steps to be converted into an active metabolite [88] and hence is less affected by variation in CYP enzymes and exerts a higher degree of platelet inhibition as compared to clopidogrel. Finally, ticagrelor, the first of a new class of P2Y_12_ inhibitors named cyclopentyl-triazole-pyrimidines, is a reversible P2Y_12_ receptor inhibitor that does not need hepatic metabolism and accordingly has a more predictable metabolic pathway resulting in a better inter-individual consistency among patients and clinical efficacy. Both prasugrel and ticagrelor have demonstrated greater efficacy than clopidogrel [86,87] and accordingly are recommended over clopidogrel in clinical guidelines in patients with no high bleeding risk [83].

Another known antiplatelet target is the GPIIb/IIIa, the most abundant platelet receptor mainly involved in platelet aggregation [89]. Two GPIIb/IIIa receptor blockers have been approved for intravenous clinical use in STEMI patients, including tirofiban (tyrosine-derived non-peptide derivative) and eptifibatide (heptapeptide). Both antagonize the GPIIb/IIIa receptor preventing fibrinogen and Von Willebrand factor (VWF) from binding to the receptor [90].

In the setting of strokes, antiplatelet therapy is used in secondary prevention in patients with non-cardioembolic transient ischemic attack or stroke. Single antiplatelet therapy with ASA or clopidogrel, or the combination of low dose ASA and dipyridamole or cilostazol, is usually recommended for secondary prevention. In some patients, a combination of ASA and clopidogrel is recommended for up to 90 days to reduce early recurrences [91]. Recent data have demonstrated that ticagrelor on top of ASA reduces the total burden of disability owing to ischemic stroke recurrence compared to ASA alone [92]. Based on these recent findings, the combination of ASA and ticagrelor for up to one month might be considered in patients at risk of ischemic stroke [91]. Cilostazol may also be used for secondary stroke prevention, particularly in Asian patients [93], since randomized clinical trials are still needed to determine its usefulness in non-Asian populations.

There are no reversal agents for the antiplatelet drugs presently used in the clinical setting. However, this might change in short/medium term for ticagrelor. Bentracimab (PB2452) is a recombinant human monoclonal antibody antigen-binding fragment with a dual mechanism of action; it binds both to free ticagrelor and to its major active metabolite (AR-C124910XX) [94]. Bentracimab is currently being tested in a phase III clinical trial (NCT04286438) in patients with uncontrolled major or life-threatening bleeding or requiring urgent survey or invasive procedure.

**Figure 3:**
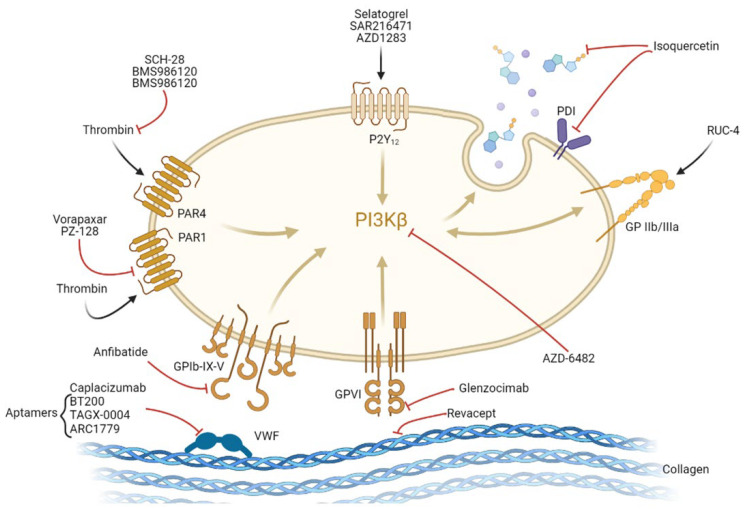
Emerging antiplatelet targets and drugs. PDI: phosphodiesterase; VWF: Von Willebrand factor; GP: glycoprotein; PAR: protease activator receptor.

Despite the currently available antiplatelet armamentarium, recurrent thrombotic events still occur, and enhanced bleeding risk remains a challenge that needs to be addressed. These limitations have stimulated research interest in identifying and developing new antiplatelet targets (Figure 3).

### 3.1. Targeting Platelet Adhesion

#### 3.1.1. Inhibition of Von Willebrand Factor-Glycoprotein 1bα-Mediated Platelet Activation

VWF is synthesized by endothelial cells and megakaryocytes. VWF activity depends on the size of the multimer being ultra-large VWF multimers highly reactive with platelets. The monomeric VWF displays a multi-domain structure which includes an A3 domain (interacts with exposed vascular collagen) and an A1 domain (binds to platelet GPIbα). A1 interaction with GPIbα favours platelet rolling and adhesion, especially under high share rate conditions. GPVI and integrin α2β1 further support tight platelet adhesion. The resultant platelet activation induces the conformational change of GPIIb/IIIa, which favours platelet–platelet interaction (i.e., platelet aggregation) by binding to fibrinogen (primary ligand) or to the C1 domain of VWF. Although VWF also plays a pivotal role in platelet aggregation by serving as an intercellular bridge between platelets, efforts have mainly focused on the discovery of pharmacological agents able to interfere with VWF-mediated platelet adhesion either by blocking the VWF-collagen or the VWF-GPIbα interaction. Promising preclinical and proof-of-concept clinical trials have supported their antithrombotic potential, as described below [95].

Anfibatide is a direct GPIb antagonist purified from snake (*Deinagkistrodon acutus*) venom that prevents GPIb interaction with VWF. Intravenous administration of anfibatide in NSTEMI patients (phase Ib/IIa study) proved feasible and safe and markedly inhibited platelet aggregation without increasing the risk of bleeding [96]. A phase II trial is currently assessing its safety and efficacy in STEMI patients before primary PCI being the primary endpoint TIMI myocardial perfusion grades (NCT02495012). In the field of stroke, administering anfibatide after cerebral ischemia/reperfusion injury in rats has been shown to significantly improve ischemic lesions alleviating inflammation and apoptosis in a dose-dependent manner [97] and preserving blood-brain barrier integrity [98]. These observations further support the contribution of platelets to inflammation and immune responses in ischemic damage beyond their function in hemostasis [99].

Caplacizumab (formerly ALX-0081) is a humanized monoclonal nanobody that targets the A1 domain of VWF, preventing its interaction with GPIb. After promising results in phase I studies (healthy subjects and stable angina patients undergoing PIC) [100], a phase II study in high-risk patients with ACS undergoing PCI (NCT01020383) is currently underway and aims to compare the safety and efficacy of caplacizumab vs. abciximab on top of standard antithrombotic therapy (ASA, clopidogrel, and heparin).

Aptamers have also been developed to block the A1 domain (Table 5). As such, BT200 has been shown to effectively block VWF activity in both ACS [101] and stroke [102] patients by binding to the VWF-A1 domain and is currently being tested in healthy volunteers (phase I, NCT04103034).

#### 3.1.2. Glycoprotein VI: Inhibition of Collagen-Mediated Platelet Activation

GPVI is a platelet- and megakaryocyte-specific 60–65 kDa immunoglobulin-like transmembrane receptor. It is expressed at the platelet surface and is associated with the FcR γ (Fc receptor γ)-chain, which is responsible for the signaling via its immunoreceptor-tyrosine-based-activation motif. GPVI is considered the main collagen receptor in platelets, although it also binds to other substrates, including fibrin, fibrinogen, fibronectin, galectin-3, or laminin [103]. Activation of the GPVI–FcRγ complex initiates intracellular signaling through a tyrosine kinase-based signaling pathway [104] that eventually triggers calcium mobilization and the resultant platelet activation [105]. Several experimental studies have supported that GPVI seems to have little or no impact on hemostasis. As such, patients lacking functional GPVI have shown mild bleeding diathesis [106] unless they have moderate to severe thrombocytopenia [107]. Likewise, a mutation in the GPVI gene identified in the Chilean population that prevents GPVI surface expression has not been associated with a significant increase in bleeding and has been hypothesized to confer a protective benefit against CVD [108]. Overall, the fact that GPVI is uniquely expressed in platelets and megakaryocytes and has reported minor involvement in hemostasis [109,110] has made GPVI inhibition a promising approach to prevent thrombosis while limiting bleeding risk.

Revacept, a competitive antagonist to GPVI-collagen signaling, is one of the most studied drugs. Revacept is a dimeric, soluble fusion protein composed of the extracellular domain of the GPVI receptor and the human Fc-fragment. It competes with endogenous platelet GPVI for binding to exposed collagen fibers preventing platelet activation [105]. Since revacept targets the exposed vascular collagen, it does not interfere with circulating platelets beyond the atherosclerotic lesion, exerting a little effect on systemic hemostasis or bleeding as suggested in experimental models and a phase I clinical trial [111]. Revacept has been tested in phase II clinical trials [112] in patients with stable coronary heart disease undergoing PCI. Yet, no significant differences were observed in the primary endpoint (death or myocardial injury) or bleeding between the treated and placebo arm. Future studies are being planned to address its efficacy in patients at higher risk of ischemic events (e.g., in the context of ACS), where collagen-induced platelet activation may play a more important role.

In the setting of ischemic stroke, revacept is currently being tested in a phase II clinical trial (NCT01645306) in patients with symptomatic carotid artery stenosis (history of ischemic stroke, transitory ischemic attack or amaurosis fugax within the last 30 days) to check its efficacy in secondary prevention of thromboembolic ischemic events.

Several monoclonal antibodies against GPVI have also been developed, as detailed in Table 5, the most important being glenzocimab (ACT017). This monoclonal antibody binds to human GPVI and has inhibited platelet adhesion, aggregation, and thrombus formation onto collagen surface under arterial flow conditions [113]. Glenzocimab has a short plasma half-life requiring to be infused intravenously for 6 or 12 h to maintain the necessary duration of effect [114]. Glenzocimab has been demonstrated to sufficiently block collagen-induced platelet aggregation in a phase I study [115] with an excellent safety profile (no evidence of thrombocytopenia or excess bleeding). Glenzocimab is being tested in a phase II/III trial to evaluate the safety and efficacy of a single dose of glenzocimab used in combination with standard of care (thrombolysis and thrombectomy) for acute ischemic stroke (ACTIVASE NCT05070260).

**Table 5:** Novel antiplatelets in the preclinical phase.

Antiplatelet	Type	Mechanism of Action	Studies Conducted So Far
**TAGX-0004 (*studies* in vitro)**	Aptamer	VWF inhibition	It has excellent affinity with VWF-A1 domain and a superior antithrombotic potential than ARC1779 [116].
**ARC1779 *(intravenous)***	Aptamer	VWF inhibition	In a phase II trial, it reduced cerebral thromboembolism in patients undergoing carotid endarterectomy [117]. However, the study was terminated due to a lack of funding and associated increased bleeding risk. Further development of ARC1779 was halted.
**AJW200 *(intravenous)***	Monoclonal antibody	VWF inhibition	Tested as adjunctive therapy with tPA in a mouse model of embolic stroke where it showed a synergistic effect and improved behavioural function [118]. In monkeys, it has been shown to inhibit high-shear-stress-induced platelet adhesion, aggregation, and thrombin generation [119].
**82D6A3 *(intravenous)***	Monoclonal antibody (A3 domain)	VWF inhibition	It has been tested in baboons, showing potent antithrombotic activities without significantly prolonging the bleeding time [120].
**Caplacizumab *(intravenous/subcutaneous)***	Nanobody	VWF inhibition	Approved for the treatment of immune-mediated thrombotic thrombocytopenic purpura [121].
**h6B4-Fab *(intravenous)***	Monoclonal antibody	GPIb inhibition	Reduce thrombus formation in baboons with minimal effect on bleeding time [122].
**SZ2 *(intravenous)***	Monoclonal antibody	GPIb inhibition	In vitro, functional studies revealed that it prevents platelet adhesion to VWF under high-shear stress and inhibits ristocetin-induced platelet aggregation in a dose-dependent manner [123].
**JAQ1 *(Intravenous)***	Monoclonal antibody	GPVI inhibition	It protects against lethal thromboembolism in mice with minimal impact on hemostasis [124,125].
**SCH-28 (*studies *in vitro)**	Small molecule	PAR4 inhibition	It inhibits PAR-4-mediated platelet activation and aggregation by blocking the thrombin exosite II binding domain [126].
**HPW-RX40 *(intravenous)***	Small molecule	PDI inhibition	Reduces thrombus formation in whole human blood under flow conditions and protects mice from ferric chloride-induced thrombus formation [127].
**ML359 (*studies* in vitro)**	Small molecule	PDI Inhibition	It exerts no cytotoxicity in three human cell lines and inhibits platelet aggregation [128].
**ML355 *(oral)***	Small molecule	12-Lipoxygenase inhibition	It reduces thrombus growth and vessel occlusion in a mouse model of arterial thrombosis with minimal impact on hemostasis [129].
**MIPS-9922 *(intravenous)***	Small molecule	PI3Kβ inhibition	It prevents arterial thrombus formation in an in vivo electrolytic mouse model of thrombosis with minimal impact on hemostasis [130].
**scFv *(intravenous)***	Antibody	GPIIb/IIIa inhibition	It has demonstrated comparable antithrombotic efficacy to currently used GPIIb/IIIa inhibitors (tirofiban and eptifibatide) in a mice model of ferric chloride-induced thrombosis with minimal impact on hemostasis [131].
**mP_6_ *(intravenous)***	Péptide	GPIIb/IIIa inhibition	It has proven superior to aspirin and is similar to ticagrelor in a mice model of ferric chloride-induced thrombosis with minimal effects on hemostasis [132].
**SAR216471 *(oral)***	Small molecule	P2Y_12_ Inhibition	It has shown potent antithrombotic activity in a rat arterio-venous shunt model with no effect on hemostasia [133].
**AZD1283 *(oral)***	Small molecule	P2Y_12_ Inhibition	It has shown potent antithrombotic efficacy in a rat model of ferric chloride_-_induced thrombosis and lowers bleeding risk compared to clopidogrel [134].
**BMS-884775 *(oral)***	Small molecule	P2Y_1_ Inhibition	It has demonstrated, in a rabbit model of thrombosis, similar efficacy to prasugrel with less bleeding risk [135].
**MRS2500 *(intravenous)***	Small molecule	P2Y_1_ Inhibition	It prevents carotid artery thrombosis in monkey models of electrolytic-mediated arterial thrombosis with a concomitant mild prolongation in bleeding time [136].
**GLS-409 *(intravenous)***	Small molecule	P2Y_1_ and P2Y_12_ Inhibition	A It attenuates thrombosis in a canine model of unstable angina and reduces platelet aggregation to a comparable extent to cangrelor or the combination of cangrelor with a selective P2Y1 inhibitor [137].
**Troα6 and Troα10 *(intravenous)***	Peptides	GPVI inhibition	It inhibits collagen-induced platelet aggregation and thrombus formation in a ferric chloride_-_induced thrombosis model without prolonging bleeding time [138].
**BI1002494 *(oral)***	Peptide	GPVI inhibition	It reduces infarct sizes and improves neurological outcomes in a mouse model of cerebral ischemia without affecting hemostasis [139].

Tyrosine kinase inhibitors are also being developed to prevent downstream signaling initiated by activation of the GPVI-FcRγ complex. As such, platelet activation through GPVI relies on a potent protein tyrosine kinase cascade culminating in the activation of the tyrosine kinase Syk (spleen-associated tyrosine kinase). Tyrosine kinase inhibitors have been shown to exert antiplatelet effects in cancer patients (e.g., pazopanib in patients with renal cell carcinoma) [140] and short-term studies with ibrutinib analogs Btki (Bruton’s tyrosine kinase inhibitors) 43607 and Btki 43761 have shown a dramatic reduction in collagen-induced platelet aggregation in non-human primates without measurable effects on plasma clotting times or bleeding risk [141]. In addition, the Syk inhibitors PRT-060318 and BI1002494 have been shown to reduce thrombus stability in vitro [142] and thrombosis in a mouse model of cerebral ischemia [139], respectively. Finally, ibrutinib has also been shown to block CLEC-2-mediated platelet activation [143]. CLEC-2 is a platelet-activating type II transmembrane receptor which has a function similar to that of GPVI in activating Syk [144].

### 3.2. Targeting Platelet Activation

#### 3.2.1. PAR1 and PAR4: Inhibition of Thrombin-Mediated Platelet Activation

Thrombin activates human platelets through the protease-activated receptor (PAR)-1 and PAR-4. PARs are G protein-coupled receptors whose activation by thrombin depends on proteolytic cleavage of the N-terminal domain of the receptor, generating a new amino terminus that acts as a tethered ligand to activate the receptor. PAR-4 has shown to interact with PAR-1 and P2Y_12_, inducing sustained platelet activation, whereas PAR-1 does not interact with ADP receptors leading to an acute platelet response. Hence, blockage of the P2Y_12_ receptor may suppress PAR-4-mediated platelet aggregation, while PAR-1-mediated effects remain unaltered [145].

PAR-1 has shown a high affinity for thrombin, whereas higher thrombin levels are required to activate PAR-4. Hence, PAR-1 has become the focus of intense research as a therapeutic antiplatelet target. Vorapaxar is a competitive PAR-1 antagonist that irreversibly binds to the ligand-binding pocket on the extracellular surface of PAR-1. Based on two large phases III clinical trials (TRA 2°P–TIMI 50 [146] and TRACER [147] vorapaxar may be used on top of standard antiplatelet therapy in the secondary prevention of ischemic events in patients with a history of MI or symptomatic peripheral artery disease. Yet, vorapaxar is contraindicated in patients with a history of stroke or transient ischemic attack because it has been associated with increased intracranial bleeding. However, subgroup analyses of both trials have found that vorapaxar might be potentially beneficial in patients with previous MI, diabetes, coronary artery bypass grafting, and ischemic stroke [148,149]. So far, vorapaxar has been shown to reduce thrombus formation in post-MI patients treated with potent P2Y_12_ inhibitors [150].

PZ-128 is a pepducin inhibitor of PAR1 for patients with CAD/ACS undergoing coronary interventions. Pepducins are lipidated peptides which target the cytoplasmic surface of their cognate receptor, not affecting the ligand-binding. PZ-128 has experimentally been demonstrated to reduce acute arterial thrombosis and atherosclerotic plaque burden [151]. Furthermore, PZ-128 was recently tested in a phase II trial in NSTEMI or stable angina patients undergoing PCI and appeared to be safe, well-tolerated, and potentially reduce periprocedural myonecrosis when administered on top of standard antiplatelet therapy [152].

As per PAR-4 inhibitors, BMS-986141 has been demonstrated to reduce platelet-rich thrombus formation under a high shear rate [153] and is currently being tested in a phase IIa trial (NCT05093790). A similar drug, BMS-986120, has been recently tested with success in humans (phase I) [154] after encouraging data from preclinical studies where it has shown robust antithrombotic activity and a low bleeding profile [155,156].

#### 3.2.2. Inhibition of Phosphoinositide 3-Kinase Beta (PI3Kβ)

PI3Kβ is a lipid kinase that acts as an important mediator in the signal transduction downstream of the activation of P2Y_12_, GPIIb/IIIa, GPVI, PAR, and GPIb and plays a pivotal role in platelet aggregation and thrombus stability.

Based on the specific PI3Kβ inhibitor, TGX-221, which has only been tested in preclinical studies, a new molecule with better pharmacological properties has been developed, AZD-6482. This drug has shown in a phase I trial to moderately inhibit ADP- and collagen-induced platelet aggregation, particularly under high shear stress conditions with only mild prolonged bleeding time [157]. In another phase I study, the combination of AZD-6482 with ASA provided greater platelet inhibition compared to DAPT with ASA and clopidogrel without translating into prolonged bleeding times [158]. A new phase II trial (STARS) is planned to test the safety and tolerability of this drug in reperfusion for stroke (NCT05363397).

#### 3.2.3. Selatogrel: The New Antagonist of the P2Y_12_ Receptor

Selatogrel (ACT-246475) is a new potent, reversible, and selective inhibitor of the P2Y_12_ platelet receptor. Its efficacy and safety have already been confirmed in phase I and II clinical trials. In contrast to the currently used P2Y_12_ inhibitors (i.e., oral or intravenous administration), selatogrel is administered subcutaneously, overcoming potential pharmacokinetic limitations of other P2Y_12_ inhibitors, including the delay of absorption and lack of enteral access for administration with oral formulations; the need for intravenous access with cangrelor; or the need for metabolization (e.g., clopidogrel and prasugrel) to be ideal in the critical 3-h window during an ACS [159]. Additionally, selatogrel seems to have a lower bleeding risk profile than clopidogrel or ticagrelor. A study performed in mice showed that the stability of hemostatic seals was undisturbed in the presence of selatogrel, unlike clopidogrel or ticagrelor. The authors suggested that the mechanism underlying the differences in blood loss profiles among these P2Y_12_ receptor antagonists was related to off-target interference with endothelial and neutrophil cells and fibrin-mediated stabilization of hemostatic seals [160]. Subsequently, phase I and phase II clinical trials have confirmed that selatogrel provides sustained and reversible P2Y_12_ platelet inhibition with an acceptable safety profile [159]. A phase III clinical trial is currently underway (NCT04957719).

#### 3.2.4. New P2Y_1_ Receptor Antagonists

Besides the P2Y_12_ receptor, human platelets express another purinergic ADP receptor named P2Y_1_. The binding of ADP to P2Y_1_ initiates platelet aggregation response which may be reverted, while P2Y_12_ activation leads to irreversible platelet aggregation. Therefore, complete platelet aggregation requires a complex interplay and coactivation of both P2Y_1_ and P2Y_12_ receptors [161]. Following this assumption, several P2Y_1_ inhibitors have been developed, though so far, they have only been tested in animal models, as detailed in Table 5 [162].

### 3.3. Targeting Platelet Aggregation and Thrombus Propagation

#### 3.3.1. New Inhibitor of GP IIb/IIIa

Zalunfiban (RUC-4) is a second-generation small-molecule platelet GPIIb/IIIa inhibitor that blocks the receptor in its inactive conformation. This blockade avoids the drug-induced thrombocytopenia associated with other GPIIb/IIIa inhibitors since it prevents the exposition of epitopes that are potential targets for thrombocytopenia-related antibodies. Subcutaneous administration of RUC-4 in healthy subjects and stable coronary artery disease patients on ASA (Phase I trial) has shown a rapid (<15 min), potent (>80% reduction of platelet aggregation), and reversible (platelet function is restored after 1–2 h) platelet inhibitory effect [163]. These observations were confirmed in a phase IIa trial in the setting of STEMI [164]. Currently, RUC-4 is being tested in phase IIb trial in STEMI patients undergoing primary PCI (NCT04825743). Other GPIIb/IIIa inhibitors have been developed and are currently being tested in the preclinical setting, as detailed in Table 5.

#### 3.3.2. Inhibition of Protein Disulfide Isomerase (PDI)

PDI is an enzyme in the endoplasmic reticulum that catalyzes the modification of thiol-disulfide bonds during protein synthesis and is also expressed on the surface of multiple cells, including platelets. Four members of the PDI family of enzymes, including PDI, ERp57, ERp72, and ERp5, are secreted from activated platelets and endothelial cells at the site of vascular injury. The mechanisms by which extracellular PDI regulates platelet function remain to be determined. However, it is thought to interact with prothrombotic components, including GPIIb/IIIa, α2β1, vWF, GPIbα, and TF supporting, and thus, platelet activation, aggregation, and coagulation [165,166,167].

Quercetin flavonoids (mainly isoquercetin) are potent PDI inhibitors present in fruits and vegetables. They have been tested primarily in the field of cancer and venous thromboembolism where it has been shown, in phase II trial, to improve hypercoagulability in advanced cancer [168]. However, its potential role in the context of CVD has yet to be established [169,170]. So far, other PDI inhibitors are in the pipeline since they have been shown to exert antithrombotic effects in vitro and in experimental animal models (Table 5).

## 4. Conclusions

Despite the major advances in antithrombotic therapy accomplished over the last decades, atherothrombotic events remain a leading cause of death worldwide. The secondary prevention of both ischemic heart disease and ischemic stroke requires effective antiplatelets and anticoagulants without bleeding side effects. Research conducted over the last years has led to a deeper understanding of the molecular mechanisms regulating atherothrombosis and hemostasis, providing new targets for intervention [5,6,171,172,173]. New antithrombotic strategies have been developed and assessed in preclinical animal models, and some have already reached clinical testing. As per the coagulation cascade, new anticoagulants have focused on the intrinsic coagulation pathway to prevent ischemic coronary and cerebral events. In this regard, although the long journey from animal studies to randomized clinical trials has just started, hopefully, some of these promising strategies will reach routine clinical use, providing the patient with optimal protection against arterial thrombosis inhibition while preserving hemostasis.

## Figures and Tables

**Table 1:** Anticoagulant reversal agents in clinical use and preclinical/clinical development.

In Clinical Use [13,14,15]	
***Agent***	***Target***
**Vitamin K**	Warfarin, acenocumarol
**Idarucimab**	Dabigatran
**Andexanet alfa**	Apixaban, rivaroxavan, edoxaban
**Protamine sulfate**	Unfractionated heparin LMWH (partially)
**Prothrombin complex concentrate, fresh frozen plasma**	Non-specific prohemostatic agents
**Preclinical/** **Clinical Development**	
***Agent***	***Target***
**Aripazine (ciraparantag/PER977) (NCT04593784)** [16,17]	LMWH, fondaparinux, FXa inhibitors, dabigatran
**γ-thrombine S195A** [18]	Dabigatran
**GDFXa-α2M complex** [19]	Rivaroxaban, apixaban, dabigatran and heparins

**Table 2:** Factor XIIa inhibitors currently under development. This table includes the emerging FXIIa inhibitors and details the studies conducted so far to assess their efficacy and safety.

Factor XIIa Inhibitors	Type	Phase	Studies Conducted So Far
**Garadacimab *(subcutaneous)***	Antibody	III	Tested in patients with C1-esterase inhibitor-deficient hereditary angioedema showing a significant reduction of angioedema attacks. A dose-dependent increase in aPTT with no change in prothrombin time was also observed without increasing of bleeding events [34,35]. Currently ongoing phase III trials (NCT04656418, NCT04739059).
**3F7 *(intravenous)***	Antibody	Preclinical	Thromboprotection in ECMO without impairing the hemostatic capacity or increasing bleeding [36,37].
**9A2 and 15H8** ***(intravenous)***	Antibody	I	Both antibodies have been shown to protect against ferric chloride-induced arterial thrombosis. 15H8 prolonged the aPTT time in non-human primates and humans and reduced fibrin formation in collagen-coated vascular grafts inserted into arteriovenous shunts in non-human primates [38].
**5C12 *(intravenous)***	Antibody	Preclinical	Thromboprotection in ECMO in non-human primates [39].
**Ir-CPI *(intravenous)***	Kunitz-type serine protease inhibitor	Preclinical	It has demonstrated antithrombotic activity in: (1) venous and arterial in vitro thrombosis models; (2) arteriovenous shunt rabbit models; and (3) extracorporeal circuit [40,41]. It can interact with factors XIIa, XIa, and Kallikrein [42].
**FXII-ASO *(subcutaneous)***	Antisense oligonucleotide	Preclinical	Prolonged the time to catheter thrombotic occlusion (implanted in jugular vein) compared to control in a rabbit model of thrombosis [43].
**ALN-F12 *(subcutaneous)***	Interfering RNA	Preclinical	Dose-dependently reduced platelet and fibrin deposition in mice models of venous and arterial thrombosis models [44].
**rHA-Infestin-4 *(intravenous)***	Kazal-type serine protease inhibitor	Preclinical	Protects against arterial and venous thrombosis in mouse and rabbit models. Reduces infarct size and brain edema formation leading to better neurological scores and survival in a mouse model of stroke [45,46,47].

aPTT: activated partial thromboplastin time; ECMO: extracorporeal membrane oxygenation.

**Table 3:** Factor XIa inhibitors currently under development. This table includes the emerging FXIa inhibitors and details the studies conducted so far to assess their efficacy and safety. VTE: venous thromboembolism; AF: atrial fibrilation; aPTT: activated partial thromboplastin time; MI: myocardial infarction.

Factor XIa Inhibitors	Type	Phase	Studies Conducted So Far
**Osocimab** *** (subcutaneous)***	Antibody	II	Effective in thromboprophylaxis in patients undergoing knee arthroplasty [54].
**Abelacimab** *** (intravenous)***	Antibody	III	Effective in preventing venous thromboembolism and is associated with a low bleeding risk [55]. There are ongoing phase III trials in cancer patients to compare the effect of abelacimab relative to apixaban (NCT05171049) or dalteparin (NCT05171075) in VTE recurrence and bleeding.
**AB023 (Xisomab) *(intravenous)***	Antibody	II	Effective and secure in patients with end-stage renal disease [56]. Ongoing phase II trial to test xisomab for the prevention of catheter-associated thrombosis in patients with cancer receiving chemotherapy (NCT04465760).
**14E11** *** (subcutaneous)***	Antibody	Preclinical	In mice, 14E11 has been shown to prevent arterial occlusion induced by ferric chloride to a similar degree as that accomplished by total FXI deficiency. In baboons, it has been shown to reduce platelet-rich thrombus growth in collagen-coated grafts inserted into arteriovenous shunts [57].
**FXI-175, FXI-203 *(intravenous)***	Antibody	Preclinical	Ferric chloride-induced thrombosis was reduced in mice treated with FXI-175 and FX-203 compared to placebo-treated mice. Neither antibody caused severe blood loss assessed through the tail bleeding assay [58].
**Frunexian EP-7041a** *** (intravenous)***	Small molecule C_19_H_27_ClN_4_O_4_	II	EP-7041 was safe and well tolerated in healthy volunteers with rapid onset and offset of action and predictable dose-related increases of aPTT [59]. In addition, there is an ongoing trial in thromboprophylaxis in COVID-19 patients (NCT05040776).
**Milvexian (BMS-986177)** *** (oral)***	Small molecule C_28_H_23_Cl_2_F_2_N_9_O_2_	II	Prevention of venous thromboembolism with low risk of bleeding (phase II) [60]. In rabbits, it has demonstrated effective antithrombotic potential with limited impact on hemostasis, even when combined with aspirin [61]. A recent phase II trial (AXIOMATIC-SSP) has shown it is safe in secondary stroke prevention [62].
**Asundexian** *** (oral)***	Small molecule C_26_H_21_ClF_4_N_6_O_4_	II b	In patients with AF, it has shown low rates of bleeding as compared with apixaban [63]. It has also shown no increase in bleeding events in MI [64] and stroke [65] patients. New phase III clinical trials have been announced to test its efficacy in patients with AF (OCEAN-AF) and in secondary prevention of stroke (OCEAN-STROKE).
**BMS-962212 *(intravenous)***	Small molecule C_32_H_28_ClFN_8_O_5_	I	Tested in healthy subjects showing good tolerance, no signs of bleeding and significant changes in aPTT and FXI clotting activity [66].
**ONO-7684 *(oral)***	Small molecule C_23_H1_6_ClF_2_N_9_O	I	It strongly inhibited factor XI coagulation activity and increased activated partial thromboplastin time [67].
**BMS-654457 *(intravenous)***	Small molecule C_36_H_37_N_5_O_4)_	Preclinical	It has been shown in vitro to increase aPTT without altering prothrombin time or ADP-, arachidonic acid-, or collagen-induced platelet aggregation. In rabbit models, it has shown equivalent antithrombotic effect to that achieved by standard doses of reference anticoagulants (warfarin and dabigatran) and antiplatelet agents (clopidogrel and prasugrel) in addition to reducing bleeding time [68].
**ONO-5450598 *(oral)***	Small molecule	Preclinical	It provided a significant reduction of thrombus formation as compared to rivaroxaban in a non-human primate arteriovenous shunt model of thrombosis [69].
**BMS-262084 *(intravenous)***	Small molecule C_18_H_31_N_7_O_5_	Preclinical	Evaluated in rabbits, where it displayed antithrombotic potential in an arteriovenous-shunt model of thrombosis, and in an electrolytic-mediated carotid arterial thrombosis [70].
**FXI-ASO (ISIS416858) *(subcutaneous)***	Antisense oligonucleotide	II	Effective in thromboprophylaxis in patients undergoing knee arthroplasty [71].

**Table 4:** Factor IXa inhibitors currently under development. This table includes the emerging FIXa inhibitors and details the studies conducted so far to assess their efficacy and safety.

Factor IXa Inhibitors	Type	Phase	Studies Conducted So Far
**Pegnivacogin *(intravenous)***	RNA aptamer	II	- Phase II trial in NSTEMI patients undergoing cardiac catheterization did not show significant differences compared with heparin [75]. - A randomized clinical trial in patients undergoing percutaneous coronary intervention had to be terminated early due to severe allergic reactions [76]. - It has decreased platelet activation and aggregation in vitro [77].
**SB249417 *(intravenous)***	Antibody	I	It has demonstrated prolongation of coagulation measures in humans [78].
**TTP889 *(oral)***	Small molecule	II	It has not been shown to be effective for the extended prevention of venous thromboembolism [79].

## Data Availability

Not applicable.
